# Peracetic acid reduces *Campylobacter* spp. on turkey skin: Effects of a spray treatment on microbial load, sensory and meat quality during storage

**DOI:** 10.1371/journal.pone.0220296

**Published:** 2019-07-24

**Authors:** Rilana Bertram, Corinna Kehrenberg, Diana Seinige, Carsten Krischek

**Affiliations:** 1 Institute of Food Quality and Food Safety, University of Veterinary Medicine Hannover, Foundation, Hannover, Germany; 2 Institute for Veterinary Food Science, Justus-Liebig-University Giessen, Giessen, Germany; University of Messina, ITALY

## Abstract

Handling and consumption of *Campylobacter*-contaminated poultry meat is the most common cause of human campylobacteriosis. While many studies deal with interventions to reduce *Campylobacter* spp. on chicken carcasses, studies on other poultry species are rare. In the present study, a spray treatment with peracetic acid (PAA) on turkey carcasses was evaluated. For this, parts of breast fillets with skin and *Campylobacter* (*C*.) *jejuni* DSM 4688 (10^8^ cfu/ml) inoculated drumsticks were sprayed for 30 s with PAA (1200 ppm) or water as control solution. Samples were packaged under modified atmosphere and stored at 4°C until analysis on day 1, 6 and 12. The breast fillets were used for determination of the total viable count, sensory and meat quality examination as well as myoglobin content and biogenic amines. The drumsticks were used for *C*. *jejuni* counts. PAA had a significant effect in reducing total viable counts on all days by up to 1.2 log_10_ compared to the untreated control. Treatment with water alone showed no effect. *C*. *jejuni* counts were significantly reduced by PAA (0.9–1.3 log_10_), while water achieved a 0.5 log_10_ reduction on *C*. *jejuni* counts on day 1. No differences in sensory, pH, electrical conductivity and myoglobin content could be found. The skin of the PAA treated fillets had lower redness values than the water control on day 1, whereas on day 12 parts of the water treated muscles were lighter than the untreated control. A lower putrescine content of the water sprayed fillets in comparison to the control sample on day 12 was the only significant difference concerning the biogenic amines. Results from this study indicate that a spray treatment with 1200 ppm PAA would be a useful measure to lower the *Campylobacter* spp. counts on turkey carcasses without having a negative influence on product quality.

## Introduction

Campylobacteriosis remains the most often reported bacterial foodborne disease in the EU [1]. Thereby 25–30% of all cases are associated with handling and consumption of chicken meat [2]. Beside chicken also other contaminated poultry meat can be a source of infection. With a share of 14% of the poultry production in the EU, turkey is the second most popular poultry meat. Production of turkey meat in the EU has increased by 6.5% between 2012 and 2016 [3]. The prevalence of *Campylobacter* spp. in turkeys depends on the season and varies between different flocks. While in the study of Atanassova et al. [4] 29% of the turkey meat was tested positive for *Campylobacter* spp., Cakmak et al. [5] found thermophilic *Campylobacter* spp. in 45.6% of the turkey meat samples. A spray treatment with peracetic acid (PAA) may be an option to reduce the *Campylobacter* spp. load on turkey carcasses and furthermore to achieve a lower microbial concentration on the meat in general. PAA is already used as additive in poultry chiller tanks in the USA for many years. Because of its broad activity spectrum, rapid action and stability at low temperatures [6, 7] as well as no toxic residues on the meat [8], PAA is a suitable antimicrobial substance. A number of in vitro studies show the effectiveness of low doses of PAA against *Campylobacter* spp. and other bacterial species [9–11]. In contrast, the efficiency of PAA on poultry meat is much lower, which may be due to the matrix of skin and feather follicles, and depends on the PAA concentration and the application method. Most of the studies achieved a significant reduction of *Campylobacter* spp. on chicken carcasses and chicken skins. Bauermeister et al. [12] added PAA in a concentration of 200 ppm to the chill water for 1 h and achieved a 1.5 log_10_ reduction of *Campylobacter* spp. in comparison to chlorine as control. Smith et al. [13] tested a 60 s dip with 100 ppm and 200 ppm PAA and achieved reductions of 0.7 and 1.4 log_10_, respectively. The same concentrations applied as spray for 62 s could only reduce *Campylobacter* counts by 0.5 and 0.6 log_10_, respectively. A 20 s dip with 1000 ppm PAA reduced *Campylobacter* spp. by 2.0 log_10_ compared to the untreated control [14]. While several studies about organic acids on chicken meat have been published, no studies about other poultry species like turkey, duck and goose could be found, which assessed the effects of a treatment with PAA on *Campylobacter* spp. counts and meat quality. So the aim of this study was to examine the effects of PAA as spray application on *Campylobacter* spp. counts and total viable counts (TVC) on turkey skin, immediately after treatment and during storage. Furthermore, to identify if there are deviations in the meat quality, sensory and physicochemical parameters (color, pH, electrical conductivity) as well as myoglobin redox forms and biogenic amines were evaluated. Because spraying does not seem to be as effective as dipping, but is the favored application method [15], we chose a relatively high concentration of 1200 ppm PAA. This proved to be the most effective concentration against *C*. *jejuni* and the TVC on chicken skin in an earlier study [16].

## Materials and methods

### Ethics statement

In the study animal material from a commercial turkey slaughterhouse was used and analyzed. The slaughterhouse considered all European and German animal welfare regulations for transport, handling and slaughter of the animals.

### Preparation of the carcasses

Experiments were performed in three replications (n = 3) on three independent days. For the trial 15 turkey hens (5 turkeys x 3 replications = 15 turkeys total) were obtained from a local poultry slaughterhouse and transported to the Institute of Food Quality and Food Safety under cooled conditions. The turkeys within each replication came from the same farm. Carcasses were stored at 4°C until the next day. The turkeys were weighed (7.32 ± 1.22 kg) and dissected 48 h after slaughter to get 18 equally sized pieces of breast fillets with skin (289 ± 82 g) and 18 deboned sections from the drumsticks with skin (259 ± 79 g). At first, for analysis of the natural contamination of the carcass skin TVC and *Campylobacter* spp. were enumerated using 5 g (ca. 30 m^2^) of the skin from each carcass. By removing the skin for analysis of the natural contamination a skinless area was obtained to analyze the effect of PAA and water on some meat quality parameters not only of the skin, but also of the meat. For the microbiological analysis of the TVC, the breast fillets were left native, whereas all drumsticks were inoculated with 1 ml of a *C*. *jejuni* DSM 4688 suspension containing 1.0–2.0 x 10^8^ cfu/ml, which was pipetted and subsequently spread on the skin with a sterile L-shaped spreader (VWR International, Darmstadt, Germany). After a drying period of 20 min at room temperature both the drumsticks and the breast fillets were treated with either PAA or sterile, distilled water (control solution) as described below.

### Inoculum preparation

*C*. *jejuni* was grown on blood agar plates (blood agar base: Carl Roth GmbH & Co. KG, Karlsruhe, Germany, supplemented with 6% defibrinated horse blood: Oxoid, Wesel, Germany) at 41.5°C under microaerobic conditions (5% O_2_, 10% CO_2_ and 85% N_2_). Colonies from a 20 h agar plate were suspended in sterile saline solution (0.9% NaCl) and adjusted with a densitometer (BioMérieux Marcy-l’Étoile, France, IDN 013615) to 0.5 Mc Farland standard, which equates a concentration of 1–2 x 10^8^ cfu/ml.

### Spraying solutions

As PAA dissociates rapidly after dilution, solutions were prepared freshly before each experiment and used within 4 hours. A 5% PAA solution (Grüssing GmbH, Filsum, Germany) was diluted with sterile distilled water to achieve a concentration of 1200 ppm (pH = 3.0). Sterile distilled water was used as control solution.

### Spray treatment

Application of the solutions was performed with a manual spray gun (Universal Spritzpistole Modell W1, Alfred Schütze Apparatebau GmbH, Weyhe-Dreye, Germany), equipped with a 0.5 mm stainless steel nozzle. The surface of the fillets and drumsticks were evenly sprayed for 30 s with 3 ml ± 0.5 ml PAA or sterile distilled water as control solution from a distance of 15 cm. Native breast fillets and inoculated but untreated drumsticks were used as positive controls.

### Packaging and storage

Each sample was packaged separately in polypropylene trays (PP, ES Plastic GmbH & Co. KG, Passau, Germany) equipped with a soaker pad. Trays were sealed in a packaging machine (Multivac T100, Sepp Haggenmueller GmbH & Co. KG, Wolfertschwerden, German) with a polyethylene-ethylene vinyl alcohol-PP transparent film (Südpack, Ochsenhausen, Germany) under modified atmosphere (30% CO_2_ and 70% O_2_). Day 1 samples were analyzed directly after packaging, whereas the remaining samples were investigated on days 6 and 12 after storage at 4°C. On days 1, 6 and 12 of storage for each treatment group (PAA, water, control) two samples were examined and the results were averaged for further statistical analysis (2 samples per treatment x 3 treatments = 6 breast fillets and 6 drumsticks per day; x 3 days = 18 breast fillets and 18 drumsticks per replication; x 3 replications = 54 breast fillets and 54 drumsticks in total). The inoculated drumsticks were used for quantification of *Campylobacter* spp., the breast fillets for analysis of TVC, sensory and meat quality parameters, myoglobin redox form percentages and biogenic amines.

### Sensory analysis

Sensory analysis of the breast fillets was carried out immediately after opening the packages. Odor and appearance were evaluated by a panel of three persons according to Blacha et al. [17]. The scale for both categories ranged from 5 points (very good—no deviation in quality) to 1 point (unsatisfactory–not acceptable). The points for appearance were multiplied with 3, summated with the points for odor and then divided by 4.

### Microbial analyses

A quantity of 5 g skin of each breast or drumstick was weighed into stomacher bags (VWR) and filled up to 50 g with sterile saline solution added with peptone buffered water (0.85% NaCl, 0.1% peptone) (VWR). The 1:10 dilution was stomachered for 2 min at 230 rpm (Stomacher 400 Circulator, Seward Ldt., Worthing, United Kingdom) and further serial tenfold dilutions up to 10^−6^ were prepared. The samples from the breast fillets were used for quantitative analysis of TVC (according to ISO 4833–1:2013). In brief, a volume of 1 ml of the dilution was pipetted into a petri dish and 12–15 ml plate count agar (CM0325, Oxoid) was added followed by incubation for 72 h at 30°C. The inoculated and treated drumsticks were examined quantitatively for *Campylobacter* spp. (according to ISO 10272–1), therefore 0.1 ml of the dilution was pipetted onto CCD-Agar plates (Oxoid) and spread evenly. The plates were incubated for 48 h at 41.5°C in a microaerobic atmosphere. For both *Campylobacter* spp. and TVC numbers plates between 5 and 300 colonies were counted and results were expressed in log_10_ cfu/g skin. The limits of detection were 10 cfu/g for TVC and 100 cfu/g for *Campylobacter* spp.

### Analysis of meat quality parameters

As one aim of the study was the analysis of the PAA effect on the meat quality, various parameters were analyzed using the untreated samples and the samples after PAA and water treatment. The measurements were all performed with breast meat that was directly sprayed with PAA/water as well as with indirectly sprayed breast meat. Indirectly means the skin was sprayed and removed before measurement of the meat.

#### Color measurement

Color values of the breast skin and meat were measured with a chromameter (Minolta CR-400, Konica-Minolta GmbH, Langenhagen, Germany) was used to calculate the L* (lightness), a* (redness) and b* (yellowness) values by using an average of five determinations.

#### pH measurement

The pH was evaluated with a portable pH meter equipped with a thermometer (Knick Portamess, Knick GmbH, Berlin, Germany) and a glass electrode (InLab 427, Mettler-Toledo, Urdorf, Switzerland) at three randomly distributed localizations of the meat.

#### Electrical conductivity measurement

The Electrical Conductivity (EC) was measured using an EC meter (Matthäus GmbH & Co. KG, Noblitz, Germany) by inserting the apparatus into the center of the breast meat perpendicular to the muscle fiber direction at three different localizations and results were expressed in mS/cm.

After the measurements, from each sample a superficial layer of 0.5–1.0 cm was cut from the surface of the meat, chopped into small cubes, frozen in liquid nitrogen and stored at -80°C for analysis of myoglobin redox form percentages and biogenic amines.

### Analysis of myoglobin redox form percentages

Analysis was carried out as described by Kernberger-Fischer et al. [18] with one modification. Briefly, 7 ml phosphate buffered saline (pH 7.4) was added to 3 g frozen meat sample and homogenized on ice for 1 min at 30,000 rpm with a Polytron PT 2500 homogenizer (Kinematica GmbH, Luzern, Switzerland). Subsequently, samples were centrifuged for 30 min at 35,000x g and 4°C (Sorvall RC 5 C Plus, Thermo Scientific Langenselbold, Germany) and the supernatant was converted into semi-micro cuvettes for analysis with a spectrophotometer (Evolution 201-UV–VIS-Spectrophotometer, Thermo Scientific) at 525, 503, 557 and 582 nm. The equations modified by Tang et al. [19] were used to quantify the amounts of the myoglobin redox forms oxymyoglobin (OxyMb), metmyoglobin (MetMb) and deoxymyoglobin (DeoMb).

### Analysis of biogenic amines

The biogenic amine concentrations of spermine, putrescine, cadaverine, histamine and spermidine were analyzed using a LaChrom Elite HPLC system (VWR-Hitachi, VWR International GmbH, Darmstadt, Germany) equipped with a LiChrospher 100 RP-18 (250mm, 5μm) column (Merck KGaA, Darmstadt, Germany) and connected to a Chromaster 5440 fluorescence detector (VWR Hitachi).

Sample preparation and derivatization was performed according to Vinci et al. [20] with modifications. In brief, 5 g meat was homogenized with 10 ml perchloric acid (0.4 M, AppliChem GmbH, Darmstadt, Germany) for 1 min at 15,000 rpm with a Polytron PT 2500 homogenizer (Kinematica GmbH, Luzern, Switzerland). Samples were then centrifuged for 20 min at 4°C and 2,700 x g. Subsequently, 1 ml of the sample solution was derivatized with dansylchloride (6.6 mg/ml, AppliChem) as described by Vinci et al. [20] and filtered through a syringe filter with a 0.45μm PTFE membrane (Pall GmbH, Dreieich, Germany). Chromatographic separation was performed based on the protocol of Özoğul [21] with minor modifications. Acetonitrile (AppliChem) and HPLC grade water, obtained from Sartorius arium pro Ultrapure Water System (Sartorius AG, Göttingen, Germany), were used for gradient elution as shown in Table 1. The injection volume of the sample solutions was 10 μl. Biogenic amines were detected with an excitation wavelength at 350 nm and an emission at 520 nm. Results are expressed in μg/g meat.

**Table 1:** HPLC chromatographic profile for separation of biogenic amines.

Time (min)	Acetonitrile % (A)	HPLC water % (B)	Flow rate (ml/min)
0	40	60	1.6
5	50	50	1.8
10	60	40	2.0
15	70	30	2.0
22	40	60	1.6

### Statistical analysis

All experiments were performed in triplicate. SAS Enterprise Guide 7.15 (SAS Institute Inc., North Carolina, USA) was used for statistical analyses. The effects of the different treatments were analyzed with one-way ANOVA. Subsequently TUKEY post-hoc-test was conducted, if the F-value was lower than 0.05. Differences were considered significant at a p-value of 0.05 or lower (p≤0.05).

## Results and discussion

### Sensory analysis

The highest score that could be achieved by sensory analysis of the PAA treated samples was 5.0, composed of appearance points (weighed with a factor of 3) and odor points. Organic acids in higher concentrations can cause bleaching or a brownish discoloration and also vinegar-like off-odors when using acetic acid [22]. It is important that the treatment with antimicrobial substances does not lead to significant deviations concerning sensory attributes because this could diminish the consumers’ acceptance [23], but the concentrations mostly used rarely have an effect on sensory parameters [22]. No statistically significant differences could be detected between the treatment groups (Table 2). According to that, results from a previous study also showed no impact of a spray treatment with 1200 ppm PAA on the sensory results of chicken breast fillets [16]. Del Rio et al. reported that chicken legs dipped in 220 ppm PAA had no different color, smell and overall acceptability than water and untreated controls [24]. In this study, the sensory scores were lower over the course of storage, but with no significant differences between the treatment groups. Nagel et al. [14] also found no sensory deviations after dipping broiler carcasses in 1000 ppm PAA. In the study of Bauermeister et al. [12], the addition of 100 or 200 ppm PAA to the chill water resulted in lower flavor of the cooked breast muscles on day 1. However, it has to be taken into account that chlorine was used as control and the carcasses were chilled in the solution for 1 h. On days 7 and 15, no sensory differences were detectable [12].

**Table 2:** Sensory results and physicochemical parameters of turkey breast fillets treated with 1200 ppm peracetic acid (PAA) or water^1^.

Day of storage	Treat-ment	Sensory analysis^2^	pH direct^3^	pH indirect^4^	EC^5^ direct	EC^5^ indirect
1	Control^6^	4.9 ± 0.1	5.69 ± 0.07	5.71± 0.06	12.9 ± 0.3	12.9 ± 0.5
	Water	4.9 ± 0.1	5.72 ± 0.09	5.74 ± 0.08	12.9 ± 0.4	13.0 ± 0.1
	PAA	4.7 ± 0.2	5.72 ± 0.12	5.71 ± 0.14	14.1 ± 2.6	12.6 ± 0.5
6	Control	4.8 ± 0.2	5.74 ± 0.07	5.74 ± 0.07	12.5 ± 0.9	12.9 ± 0.2
	Water	4.7 ± 0.2	5.73 ± 0.15	5.72 ± 0.15	13.0 ± 0.3	13.0 ± 0.3
	PAA	4.5 ± 0.4	5.72 ± 0.08	5.74 ± 0.08	13.0 ± 0.2	12.9 ± 0.3
12	Control	4.2 ± 0.3	5.71 ± 0.09	5.71 ± 0.08	12.2 ± 2.2	12.5 ± 1.7
	Water	4.3 ± 0.5	5.63 ± 0.06	5.64 ± 0.07	12.7 ± 0.8	12.5 ± 1.1
	PAA	4.1 ± 0.5	5.72 ± 0.07	5.65 ± 0.08	13.1 ± 0.2	12.9 ± 0.4

^1^Least square means (LSM) and standard deviations (±SD); n = 3 per storage day

^2^Where 5 = very good—no deviation in quality; 1 = unsatisfactory–not acceptable

^3^direct = parameter determined after application of PAA directly to the meat surface

^4^indirect = parameter determined after application of PAA to the skin before analysis of the parameter on the meat below the treated skin

^5^EC = Electrical conductivity in mS/cm

^6^Untreated breast fillets served as control

### Microbial analysis

The average initial TVC contents of the turkey breast skins were 1.5 x 10^4^ cfu/g skin. Nearly all samples were initially *Campylobacter* spp. negative, except for the last trial, where *Campylobacter* spp. counts between 2.0 x 10^1^ and 1.7 x 10^3^ cfu/g skin were detected on the breast skin samples. We assume that this had no impact on the results, because drumsticks were inoculated with a much higher concentration of 1.0 x 10^8^ cfu/ml and the positive control showed no higher *Campylobacter* spp. counts than the positive controls of the formerly *Campylobacter* spp.-negative turkeys.

**Fig 1:**
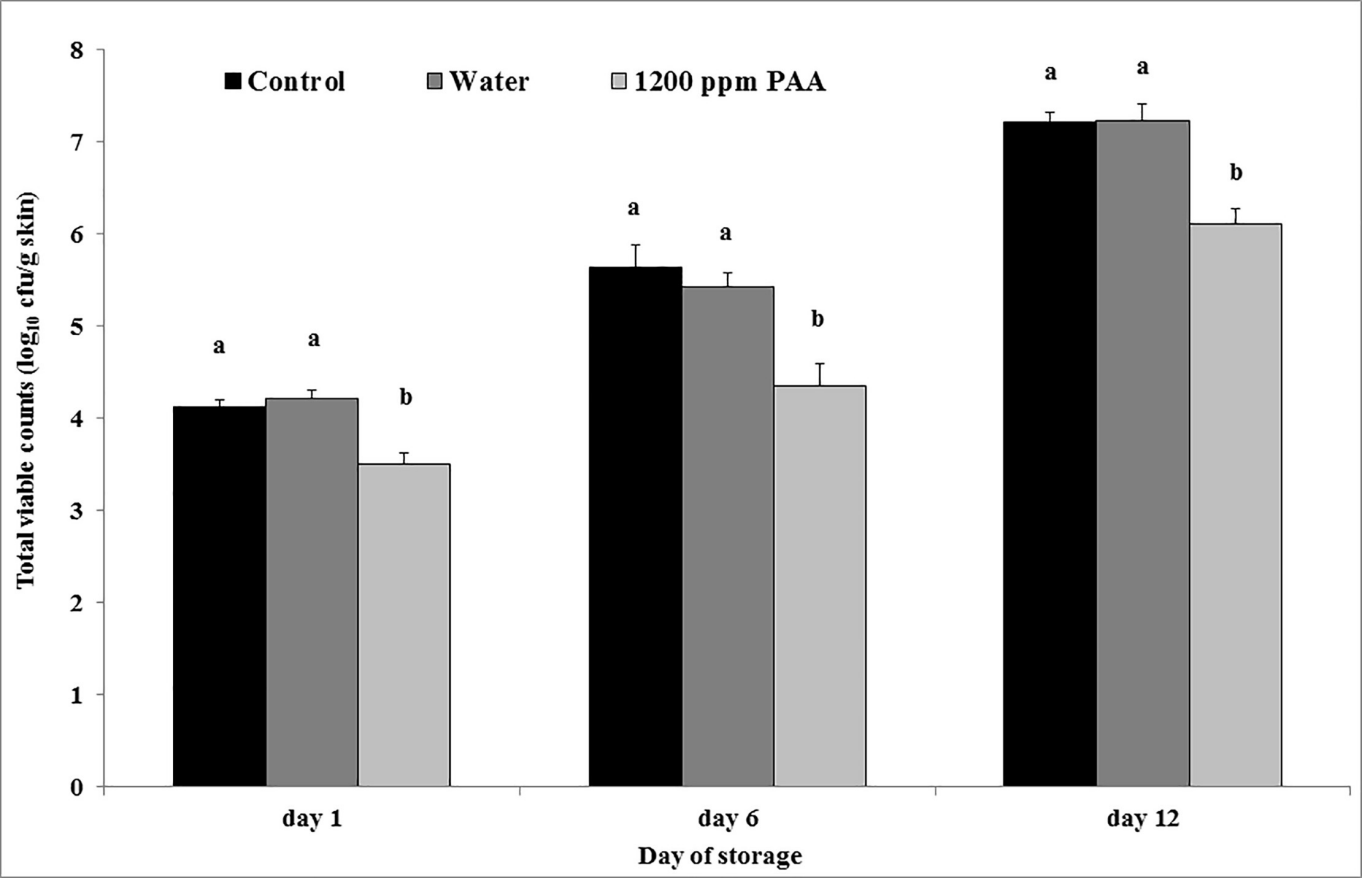
Least square means (LSM) and standard errors (SE) of the total viable counts on turkey breast skin after spray treatment. n = 3 per storage day; untreated breast skin served as control. Means with no common letter at the same day differ significantly (P ≤ 0.05).

The application of PAA on turkey meat samples showed a reducing effect on TVC in comparison to the water and control groups on all days of storage (Fig 1). The highest reduction of 1.2 log_10_ could be seen on day 6 in comparison with the untreated control. Treatment with water showed no effect. Del Rio et al. [24] achieved an average reduction of 0.5 log_10_ cfu/g skin on the mesophilic aerobic count with a 220 ppm PAA treatment of chicken skins, but a significant difference between the PAA treated and the untreated group could be detected only on the first and fifths day of storage. The highest reduction of 0.9 log_10_ cfu/g skin in relation to the untreated control could be seen on day 1. According to that, in the study of Duan et al. [25] various solutions like sodium hypochlorite, chlorine dioxide and lactic acid in different concentrations were used for a spraying treatment on broiler carcasses and similar reductions up to 0.83 log_10_ cfu/cm^2^ skin were reached.

**Fig 2:**
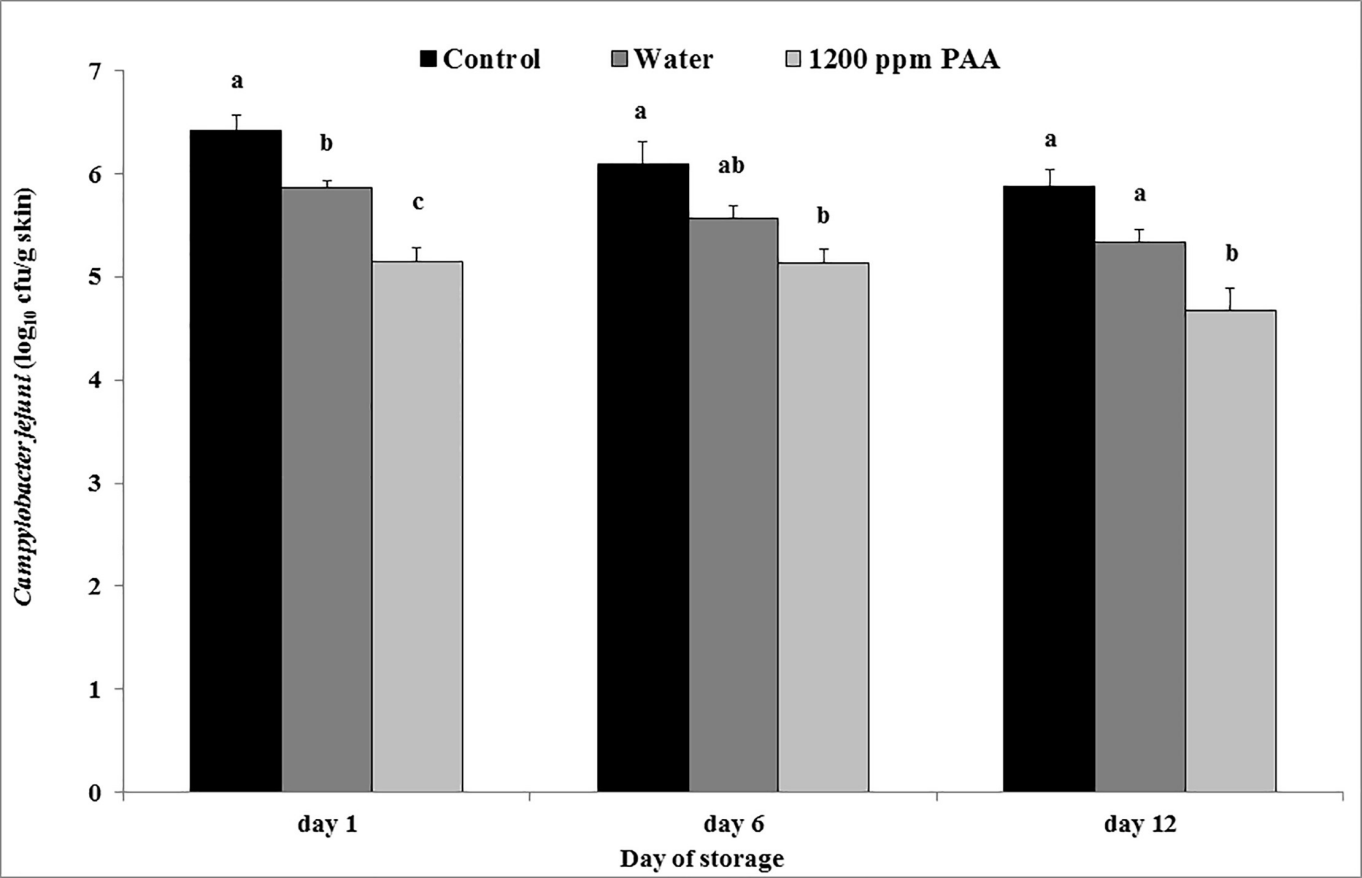
LSM and SE of the *Campylobacter (C*.*) jejuni* counts on turkey drumstick skin after spray treatment. n = 3 per storage day; inoculated but untreated drumstick skin served as control. Means with no common letter at the same day differ significantly (P ≤ 0.05).

On day 1, the water treatment of samples reduced *C*. *jejuni* by 0.5 log_10_ and the PAA treatment by 1.3 log_10_ compared with the untreated control (Fig 2). On days 6 and 12 only the treatment with PAA had a significant effect on *C*. *jejuni* counts with a 0.9 log_10_ and 1.2 log_10_ reduction, respectively. Other studies have used chicken carcasses or chicken skin and achieved similar results. Purnell et al. [26] reported the same reduction of 1.2 log_10_ cfu/g skin with a lower concentration of 400 ppm PAA by using an automated spray rig, although the differences in bacterial counts compared to the control were not significant (p<0.01). In two studies from Nagel et al. [14] and Park et al. [27] higher concentrations of 1000 and 1200 ppm PAA were used as dip treatments, which led to reductions of 2.0 log_10_ cfu/ml and 2.6 log_10_ cfu/g skin, respectively. Zhang et al. [28] used concentrations of 700 and 1000 ppm PAA in a postchill decontamination tank, both concentrations achieved a reduction of 1.5 log_10_ cfu/ml on chicken parts. A lower concentration of 200 ppm PAA as spray treatment had no significant effect on *Campylobacter* counts in another study [29]. Smith et al. [13] directly compared two application methods of PAA. They showed that at 200 ppm PAA the reducing effect was significantly higher with the immersion treatment compared to a spraying application. When comparing the two latter studies and our study the results indicate that a higher concentration of PAA is necessary to effectively reduce *Campylobacter* spp. loads on poultry carcasses with a spraying treatment.

In the present study, the water treatment resulted in a reduction of *C*. *jejuni* counts but had no effect on TVC. This might be due to a different adherence of the bacteria to the skin. The artificially inoculation with *C*. *jejuni* might possibly lead to a reduced attachment of the bacterial cells to the turkey skin compared to the natural bacterial flora of the turkey meat, which is detected here as the TVC. Therefore some *Campylobacter* cells could be washed off regardless of the used spraying solution, however, the reason still remains to be clarified. Similar effects concerning TVC and *C*. *jejuni* counts on chicken skin could be seen in the previous study of Bertram et al. [16].

### Analysis of meat quality parameters

The electrical conductivity and the pH value are valuable criteria for determining the meat quality, with EC being also an indirect parameter for drip loss [30]. Neither EC nor pH showed any significant alterations between the treatment groups (Table 2).

This is in accordance with the results of PAA-sprayed chicken fillets in a recent study [16]. While no other studies could be found which determined the EC after treatment with PAA, our results regarding pH-values were in agreement with Park et al. [27], who dipped chicken breast skin in 1200 ppm PAA and del Rio et al. [24] who dipped chicken legs in 220 ppm for 15 min. In both studies no differences were found in the pH values in comparison with the water treated and untreated groups. There was also no difference before and after treatment of chicken breast meat with PAA [27]. In contrast, Ellebracht et al. [31] observed lower pH values of beef trimmings after dipping in PAA. This difference might be due to a different method of pH determination, as they measured right on the meat surface, where the acid has its main effect, instead of inserting the pH meter in the muscle. As color is an important criteria for consumer when buying meat [23], color measurements were performed during the course of the present study. Slight differences in color were detected between control and treatment groups (Table 3). On day 1 the skin of the PAA-treated fillets had lower a*-values than the water control. On day 12 the indirect water treated breast muscles were lighter in comparison to the untreated control. However, these differences were minimal and could not be perceived at the sensory examination. In the study of Bauermeister et al. [12] the skin color of chicken carcasses, chilled for 1 h with added PAA to the chilling water, was lighter on day 7 and 15 and showed lower a* and b* values on day 1 and 15. Chen et al. [32] also found higher L* values, as well as lower a* values in PAA treated ground chicken breast meat when compared to a chlorine treatment. The study of Bertram et al. [16] instead found no significant changes in skin and meat color of chicken breast fillets after a spray treatment with 1200 ppm PAA.

**Table 3:** Color values of turkey breast meat and skin treated with 1200 ppm peracetic acid (PAA) or water^1^.

Day of storage	Treat-ment	Skin color			Meat color direct^5^			Meat color indirect^6^		
		L*^2^	a*^3^	b*^4^	L*	a*	b*	L*	a*	b*
1	Control^7^	70.5 ± 3.5	4.1 ± 1.7**ab**	3.8 ± 4.7	54.1 ± 1.5	3.0 ± 0.5	0.4 ± 0.9	54.8 ± 2.8	3.2 ± 0.7	0.3 ± 1.2
	Water	70.7 ± 1.9	4.9 ± 1.7**a**	2.6 ± 2.9	57.1 ± 3.2	2.5 ± 0.4	0.0 ± 4.4	57.2 ± 4.1	2.5 ± 0.6	0.4 ± 1.9
	PAA	69.5 ± 5.2	2.5 ± 1.0**b**	-0.9 ± 2.4	56.5 ± 3.4	2.2 ± 1.3	-1.3 ± 1.6	55.3 ± 2.6	3.0 ± 0.6	0.2 ± 0.5
6	Control	71.2 ± 2.3	2.9 ± 0.7	2.7 ± 3.3	53.9 ± 1.8	3.8 ± 0.8	0.1 ± 1.4	53.8 ± 2.0	3.8 ± 0.6	0.0 ± 0.9
	Water	73.6 ± 1.6	3.2 ± 0.7	3.0 ± 2.0	54.8 ± 2.0	3.7 ± 0.7	0.5 ± 1.7	55.2 ± 1.4	3.6 ± 0.9	0.1 ± 1.6
	PAA	70.7 ± 3.8	2.5 ± 0.7	0.5 ± 3.3	55.8 ± 2.1	3.1 ± 0.7	-0.9 ± 2.0	56.0 ± 4.2	3.7 ± 1.0	-0.67± 1.0
12	Control	70.4 ± 1.9	1.8 ± 0.9	-0.6 ± 1.6	54.1 ± 1.8	3.3 ± 0.5	0.2 ± 1.2	53.7 ± 1.8**b**	4.2 ± 1.0	0.3 ± 1.0
	Water	73.1 ± 2.0	2.4 ± 0.7	1.3 ± 2.2	57.9 ± 3.0	3.1 ± 0.7	0.5 ± 1.9	57.3 ± 2.2**a**	3.6 ± 0.6	-0.1 ± 1.2
	PAA	73.2 ± 3.6	1.7 ± 0.7	1.7 ± 3.2	56.3 ± 2.7	2.6 ± 0.7	-0.8 ± 2.3	55.9 ± 3.1**ab**	3.6 ± 1.1	-0.6 ± 1.6

^1^LSM ± SD

^2^L* = lightness

^3^a* = redness

^4^b* = yellowness

^5^direct = color values determined after application of PAA directly to the meat surface

^6^indirect = color values determined after application of PAA to the skin before analysis of the parameter on the meat below the treated skin

^7^untreated breast fillets served as control

^ab^LSM within a column with no common letter on the same day of storage differ significantly (P ≤ 0.05)

n = 3 per storage day

### Analysis of myoglobin redox form percentages

In accordance with the color values, as there were only small divergences, no significant changes in the myoglobin redox forms could be seen when comparing treatment and control groups of the present study (Table 4). When chicken breast fillets were treated with 1200 ppm PAA in a previous study [16], there was only a small impact on the myoglobin redox forms. The indirectly treated fillets showed higher OxyMb values and therefore lower DeoMb values on day 12 in comparison with the water control.

**Table 4:** Myoglobin redox form percentages of turkey breast meat treated with 1200 ppm peracetic acid (PAA) or water^1^.

Day of storage	Treat-ment	OxyMb (%)		MetMb (%)		DeoMb (%)	
		direct^2^	indirect^3^	direct	indirect	direct	indirect
1	Control^4^	27.6 ± 4.9	25.2 ± 6.6	51.5 ± 4.0	53.7 ± 5.9	20.3 ± 1.3	20.7 ± 1.1
	Water	26.4 ± 4.4	23.5 ± 5.0	52.1 ± 3.8	55.1 ± 4.6	21.1 ± 1.3	21.1 ± 0.6
	PAA	23.7 ± 8.4	22.8 ± 4.1	55.0 ± 7.1	55.9 ± 3.7	21.0 ± 1.8	21.1 ± 1.0
6	Control	24.5 ± 5.1	29.8 ± 3.0	54.8 ± 4.3	50.2 ± 2.6	20.3 ± 1.1	19.4 ± 0.7
	Water	28.6 ± 4.4	27.0 ± 5.8	50.8 ± 3.1	52.5 ± 4.5	20.0 ± 1.8	20.0 ± 1.7
	PAA	29.4 ± 3.9	28.4 ± 2.4	50.0 ± 2.6	51.2 ± 1.3	20.1 ± 1.5	19.9 ± 1.4
12	Control	22.9± 5.3	20.9 ± 4.6	54.3 ± 4.6	54.9 ± 5.0	22.67± 2.5	24.2 ± 3.6
	Water	27.2 ± 1.4	20.5 ± 5.1	51.3 ± 1.2	56.9 ± 5.5	21.1 ± 1.3	22.5 ± 2.3
	PAA	26.4 ± 4.5	23.1 ± 5.5	51.4 ± 2.5	54.1 ± 4.1	21.8 ± 2.6	22.6 ± 3.9

^1^LSM ± SD

^2^direct = parameters determined after application of PAA directly to the meat surface

^3^indirect = parameters determined after application of PAA to the skin before analysis of the parameter on the meat below the treated skin

^4^Untreated breast fillets served as control

n = 3 per storage day

Mohan et al. [33], who treated beef trimmings with 200 ppm PAA, found that the treated samples had lower a* values than the untreated inoculated control and a lower saturation index in comparison to both the untreated control and the untreated inoculated control. In contrast, the 630/580 ratio, which describes the meat color from 1 = brown to 5 = bright purplish red, had higher numerical numbers for the PAA treated samples. However, the ratio is just an approximate indication of the real oxymyoglobin content and beef trimming was used in that study, while in the present study only the surface or the skin above the muscle was sprayed.

### Analysis of biogenic amines

Biogenic amines can be considered as a freshness marker for meat [20] and were therefore investigated to see if PAA affects the shelf life of turkey meat. Results are presented in Table 5. Peracetic acid did not have any significant effects on the content of the five biogenic amines. The only significant difference was a lower putrescine content of the indirect water treated samples in comparison with the control on day 12. The high variation in the data may be explained through an insufficient number of statistically evaluable measurements. As the method is very sensitive many of the measured values were not analyzable because of background noises due to the matrix of the meat. Some also fell under the limit of quantification. Min et al. [34] showed that a treatment with organic acids like acetic, citric and lactic acid could reduce the total amount of biogenic amines on inoculated ground chicken, but this effect depended on the inoculated organisms and the used organic acid. Some values of single amines were actually higher than in the control group.

**Table 5:** Biogenic amines of turkey breast meat treated with 1200 ppm peracetic acid (PAA) or water^1^.

Day of storage	Treat-ment		Spermine (μg/g)	Putrescine (μg/g)	Cadaverine (μg/g)	Histamine (μg/g)	Spermidine (μg/g)
1	Control^4^	direct^2^	26.1 ± 25.2	1.9 ± 1.4	1.2 ± 0.4	1.3 ± 1.4	37.0 ± 38.2
		indirect^3^	23.3 ± 20.7	1.7 ± 1.1	1.0 ± 0.0	6.5 ± 13.2	43.5 ± 37.4
	Water	direct	26.1 ± 27.1	2.1 ± 1.3	1.4 ± 0.6	3.9 ± 5.2	31.8 ± 33.4
		indirect	13.6 ± 8.7	1.8 ± 1.3	1.2 ± 0.4	3.0 ± 4.8	21.6 ± 22.9
	PAA	direct	20.4 ± 17.6	2.0 ± 1.3	1.2 ± 0.4	12.1 ± 18.2	43.3 ± 31.7
		indirect	18.1 ± 19.8	2.3 ± 1.6	1.2 ± 0.5	4.5 ± 6.3	43.1 ± 37.2
6	Control	direct	21.6 ± 20.6	2.0 ± 1.1	1.2 ± 0.4	1.3 ± 1.5	31.1 ± 27.1
		indirect	25.0 ± 24.6	2.4 ± 1.5	1.3 **±** 0.6	1.2 ± 1.9	44.1 ± 24.5
	Water	direct	21.0 ± 18.6	3.2 ± 2.3	1.4 ± 0.6	1.2 ± 1.2	36.7 ± 41.3
		indirect	18.9 ± 16.2	3.0 ± 1.1	2.2 ± 0.6	3.5 ± 4.5	41.5 ± 49.6
	PAA	direct	25.1 ± 24.5	2.6 ± 1.3	1.4 ± 0.6	3.2 ± 3.7	22.8 ± 32.0
		indirect	25.7 ± 24.4	2.4 ± 1.3	1.5 ± 0.6	2.5 ± 2.4	33.7 ± 35.9
12	Control	direct	9.9 ± 0.04	7.7 ± 7.8	7.1 ± 10.5	2.6 ± 1.6	17.0 ± 21.4
		indirect	76.7 ± 163.5	14.2 ± 9.1**a**	8.3 ± 13.9	4.3 ± 3.8	35.4 ± 24.2
	Water	direct	23.9 ± 21.7	5.2 ± 2.4	5.8 ± 6.9	2.4 ± 1.8	25.0 ± 32.9
		indirect	10.0 ± 0.03	5.6 ± 2.0**b**	15.6 ± 13.2	2.5 ± 2.3	22.7 ± 24.1
	PAA	direct	62.8 ± 129.5	5.0 ± 4.4	1.6 ± 0.7	5.2 ± 4.4	23.8 ± 28.9
		indirect	24.5 ± 23.5	6.1 ± 3.3**ab**	5.1 ± 8.4	0.9 ± 0.7	43.0 ± 34.0

^1^LSM ± SD

^2^direct = parameters determined after application of PAA directly to the meat surface

^3^indirect = parameters determined after application of PAA to the skin before analysis of the parameter on the meat below the treated skin

^4^Untreated breast fillets served as control

n = 3 per storage day

^ab^LSM within a column with no common letter on the same day of storage and the same group(direct/indirect) differ significantly

Storage under MAP can also influence the amount of biogenic amines. Fraqueza et al. [35] found a significant lower content of putrescine, cadaverine and spermidine in different mixtures of MAP than in aerobic packaged turkey meat on day 12 of storage. Tyramine and spermine instead were not significantly different.

## Conclusions

The results of the present study show, that PAA can be applied as spray treatment on turkey carcasses to reduce *Campylobacter* spp loads, although only a reduction up to 1.3 log_10_ cfu/g could be achieved. However, these reductions are in accordance with other studies using chicken carcasses. Slight differences in color on single days were the only deviations in quality, which could not even be perceived in the sensory analysis. Furthermore, a lower TVC could be achieved, which lasted during 12 days of storage. Further studies are required to test the effectiveness of the method in routine practice.
